# Automated identification of clinical features from sparsely annotated 3-dimensional medical imaging

**DOI:** 10.1038/s41746-021-00411-w

**Published:** 2021-03-08

**Authors:** Nadav Rakocz, Jeffrey N. Chiang, Muneeswar G. Nittala, Giulia Corradetti, Liran Tiosano, Swetha Velaga, Michael Thompson, Brian L. Hill, Sriram Sankararaman, Jonathan L. Haines, Margaret A. Pericak-Vance, Dwight Stambolian, Srinivas R. Sadda, Eran Halperin

**Affiliations:** 1 Department of Computer Science, University of California, Los Angeles, CA USA; 2 Department of Computational Medicine, University of California, Los Angeles, CA USA; 3 Doheny Eye Institute, Los Angeles, CA USA; 4 Department of Ophthalmology, David Geffen School of Medicine, University of California, Los Angeles, CA USA; 5 Faculty of Medicine, Hebrew University of Jerusalem, Department of Ophthalmology, Hadassah-Hebrew University Medical Center, Jerusalem, Israel; 6 Department of Human Genetics, University of California, Los Angeles, CA USA; 7 Department of Population & Quantitative Health Sciences, Case Western Reserve University, Cleveland, OH USA; 8 John P. Hussman Institute for Human Genomics, University of Miami Miller School of Medicine, Miami, FL USA; 9 Department of Ophthalmology, University of Pennsylvania, Perelman School of Medicine, Philadelphia, PA USA; 10 Department of Anesthesiology, University of California, Los Angeles, CA USA; 11 Institute of Precision Health, University of California, Los Angeles, CA USA

**Keywords:** Machine learning, Three-dimensional imaging, Biomarkers, Eye manifestations

## Abstract

One of the core challenges in applying machine learning and artificial intelligence to medicine is the limited availability of annotated medical data. Unlike in other applications of machine learning, where an abundance of labeled data is available, the labeling and annotation of medical data and images require a major effort of manual work by expert clinicians who do not have the time to annotate manually. In this work, we propose a new deep learning technique (SLIVER-net), to predict clinical features from 3-dimensional volumes using a limited number of manually annotated examples. SLIVER-net is based on transfer learning, where we borrow information about the structure and parameters of the network from publicly available large datasets. Since public volume data are scarce, we use 2D images and account for the 3-dimensional structure using a novel deep learning method which tiles the volume scans, and then adds layers that leverage the 3D structure. In order to illustrate its utility, we apply SLIVER-net to predict risk factors for progression of age-related macular degeneration (AMD), a leading cause of blindness, from optical coherence tomography (OCT) volumes acquired from multiple sites. SLIVER-net successfully predicts these factors despite being trained with a relatively small number of annotated volumes (hundreds) and only dozens of positive training examples. Our empirical evaluation demonstrates that SLIVER-net significantly outperforms standard state-of-the-art deep learning techniques used for medical volumes, and its performance is generalizable as it was validated on an external testing set. In a direct comparison with a clinician panel, we find that SLIVER-net also outperforms junior specialists, and identifies AMD progression risk factors similarly to expert retina specialists.

## Introduction

The application of deep learning, specifically Convolutional Neural Networks (CNNs), has proven to be successful for detecting and predicting disease from medical image data^1–5^. However, the application of deep learning to novel tasks has been hampered by the availability of appropriately annotated training data. Biomedical research questions, in particular, present an inherent challenge in terms of sample size. While large datasets have been released in collaboration with medical imaging (e.g., CheXpert^6^ (224,316 X-rays), ISIC^7,8^ (25,331 dermoscopic images), ABCD-NP^9^ (8500 MRI volumes), and others, e.g., http://www.grand-challenge.org/), current regulations (e.g., HIPAA in the United States) restrict the ability to collect sufficient data to apply deep learning to novel questions. Generally, clinical and biomedical research reports are based on small cohorts numbering in hundreds. For example, Lutkenhoff et al.^10^ established the largest annotated cohort of patients (143) with disorders of consciousness, and the ImageCLEF initiative curated 403 CT scans for the study of tuberculosis^11^. In addition, Lei et al.^12^ analyzed 138 patients to determine the risk for age-related macular degeneration. In addition to the extensive clinical time required to collect cohorts, there is the added burden of manually annotating patient information to enable machine learning^1,13–15^. All these factors present a high cost for applying deep learning methods to new data modalities and address novel questions.

Transfer learning^1,13,16,17^ can be used to address the small number of annotated (or labeled) samples by introducing information from another domain. However, when the data consists of 3-dimensional volumes, transfer learning cannot be directly applied unless other 3-dimensional volumes are available in sufficient quantity for reference in external datasets. Unlike resources for 2-dimensional images such as ImageNet^18^, no such resource is available for 3-dimensional data (e.g., CT, MRI, OCT, etc.). To circumvent this problem we developed a protocol for applying deep learning to a dataset with limited annotated 3-dimensional imaging data. Our approach leverages external datasets of 2-dimensional images and uses transfer learning to predict AMD-related biomarkers in 3-dimensional volumes. We transformed 3-dimensional to 2-dimensional data to make it compatible with the external set. Converting 3-dimensional to 2-dimensional data results in loss of information, therefore, we introduced an operation (slice integration) to counter the information loss. We name this approach SLice Integration of Volumetric features Extracted by pre-trained Residual neural networks (SLIVER-net).

To illustrate the effectiveness of SLIVER-net, we tested the ability of SLIVER-net to identify risk factors for retinal disease from optical coherence tomography (OCT) images. Because of its high axial resolution and histological detail, OCT is able to assess the integrity of the retinal layers^19–22^ in a variety of conditions including optic nerve disorders^23^, retinal diseases^24^, and systemic conditions which may have ocular manifestations^25,26^. OCT has been particularly transformative in the management of age-related macular degeneration (AMD), the leading cause of blindness in developed nations. Initially, AMD may manifest drusen, which are accumulations of material under the retinal pigment epithelium (RPE). Vision may be relatively good at this early or intermediate stage. Eventually, a significant number of patients develop macular neovascularization (MNV) and/or geographic atrophy (GA), which are considered late manifestations and associated with considerable loss of vision. Effective treatments (anti-vascular endothelial growth factor, or anti-VEGF) have been developed for MNV, but thus far, there is no treatment for GA. In addition, despite the availability of treatments for MNV, many “successfully” treated patients eventually go on to develop atrophy and vision loss. The best outcomes for the treatment of active MNV are observed in patients who are treated early while the neovascular lesions are small. Therefore, identifying patients who are at high-risk for progression to late AMD is essential to identify appropriate intervals for monitoring patients with earlier stages of AMD. A number of OCT risk factors for progression to late AMD have been defined and include intraretinal hyperreflective foci (which are thought to represent migration of RPE into the retina), hyporeflective cores within drusen (shown to correspond to calcific nodules^27^), subretinal drusenoid deposits, and high central drusen volume. Recently, Lei et al.^12^ proposed a system using OCT images for integrating these factors into a simple score that could reflect a given patient’s risk for conversion to late AMD. This system was later validated by Nassisi et al.^28^ in a post hoc analysis of intermediate AMD fellow eyes from subjects enrolled in the HARBOR study. Despite this compelling data regarding these OCT biomarkers which could be used to risk stratify patients and define appropriate intervals for monitoring, most clinicians do not have time to assess these OCT features in the context of a busy clinical practice. Ideally, these risk factors for progression should be detected automatically from the OCT, which would allow a risk score to be immediately available to the clinician. Such a risk score could also potentially be used to identify high-risk patients for enrollment into early intervention trials or to monitor disease progression over time in a more precise or quantitative fashion. Moreover, beyond its immediate clinical impact, an automated system to assess risk on OCT could be used for research investigations to probe large datasets such as the UK Biobank or the electronic health records and image databases of large health systems. This would allow the variability of the evolution of these biomarkers to be more precisely characterized. An automated risk score could also be used as a quantitative endophenotype in genetic discovery studies, particularly those aimed at identifying genetic risk factors for disease progression.

We applied SLIVER-net to automatically identify these factors, henceforth termed “biomarkers”. Recent applications to OCT images have focused on predicting glaucoma^29,30^, different severities of AMD^31^, and other diseases^4,32,33^. Because the clinical and biological bases for these biomarkers are still under investigation, there are relatively few examples with which we can develop a deep learning approach. SLIVER-net specifically targets such scenarios, in which the number of annotated 3-dimensional images is small (in the hundreds). Still, SLIVER-net was able to outperform current methods and sometimes better than the retina specialists. Our results demonstrate that our method is superior to expert retinal image graders. Notably, the improvements provided by SLIVER-net are primarily driven by transfer learning and slice integration, both of which are not limited to biomarker prediction nor OCT classification, and thus applicable to other 3-dimensional imaging modalities. Our analysis was done on a few hundred annotated images and demonstrates the utility of SLIVER-net for analyzing a small dataset and generalizing the annotation for a larger database.

## Results

### The SLIVER-net model

Our model, SLIVER-net, is a novel deep neural network architecture designed to operate on 3-dimensional images despite a limited number of manually annotated examples. In order to cope with the small sample size of labeled data SLIVER-net leverages external information through transfer learning from 2-dimensional images, then fine-tuned using a small set of labeled 3-dimensional images (with medically relevant annotations). The labels of the 2-dimensional images are not required to have any medical relevance, as previous investigations have shown that models learn to represent domain-general features in the transfer learning paradigm^17^. Typically, the 3-dimensional volumes with desired labels can number in the hundreds, while the external dataset will consist of tens of thousands, or ideally millions of images. After training SLIVER-net can be applied to a 3-dimensional image to predict the annotated outcomes without further need of the external dataset.

To enable transfer learning between images and volumes SLIVER-net differs from standard algorithms in two ways. First, it re-frames the 3D OCT volume as a 2D “tiling” (e.g., mosaic) of slices, allowing for the use of transfer learning with currently available 2-dimensional datasets. Second, there are additional layers to the deep neural network which enable SLIVER-net to preserve the 3-dimensional spatial structure lost by tiling (see the “Methods” section: Table 2 for further details).

The SLIVER-net model itself consists of three steps. First, the re-framed OCT volume (tiled images) is passed through a “backbone” convolutional neural network (CNN), for which the output is a representation in an abstract feature space. Then, a slice aggregation operation is applied to compress this representation and obtain information that is shared across adjacent slices. Finally, a decision module operates on this compressed representation to determine the presence or absence of biomarkers. A more detailed description of SLIVER-net is provided in the “Methods”.

### AMD-related biomarker prediction

In order to demonstrate its utility, we applied SLIVER-net to biomarker prediction from OCT, which has been the primary driver of breakthroughs in the understanding and characterization of novel biomarkers associated with AMD^34^. The identification of these biomarkers in an OCT scan requires careful manual inspection and annotation of each slice (termed a B-scan) within the OCT volume, which is highly laborious and time-consuming. It is therefore desirable to develop automatic tools that will replace manual annotation. Thus, we developed SLIVER-net to automatically predict biomarkers in early and intermediate AMD.

Data were collected across three sites: University of Miami (369 patients), Case Western Reserve University (248 patients), and University of Pennsylvania (390 patients). We employed an external validation approach, where data from two of the sites, the University of Miami and Case Western Reserve University, were used to develop and validate the model, and data from the University of Pennsylvania were reserved as an external testing set (see “Methods” for additional details). The separation into three different datasets ensured that there was no overlap between the patients used for model development and testing. In total, the training and testing sets included OCT volumes from 1007 patients, which is currently the largest available dataset annotated for these biomarkers^34^.

In order to overcome the challenge of a limited dataset, we incorporated a large publicly available dataset^4^, containing 84,495 2-dimensional OCT images (only horizontal B-scans passing through the foveal center) using transfer learning. These 2-dimensional fovea-centered OCT images provide only partial information since they do not contain 3-dimensional volume information and no information about macular regions beyond the foveal depression. This scenario fits the case for which SLIVER-net was designed. We trained SLIVER-net using this external information from 2-dimensional fovea-centered scans, along with the 3-dimensional information from the OCT volumes from the University of Miami and Case Western Reserve University.

SLIVER-net was successfully able to predict the four AMD-related OCT biomarkers evaluated in this study. Three of these biomarkers, intraretinal hyperreflective foci, subretinal drusenoid deposits, and hyporeflective drusen cores, were manually annotated, while the other biomarker (high central drusen volume) was determined based on information provided from another OCT device (Cirrus OCT). In addition, SLIVER-net was able to use the OCT data alone to predict another marker (reticular pseudodrusen) determined by infrared reflectance.

### Comparison of SLIVER-net to state-of-the-art deep learning approaches

We compared SLIVER-net with two alternative models: a 3D CNN and a 2D CNN using the same image stacking approach. 3D CNNs, which are commonly used for MRI and CT analysis^35–37^, represent the current state of the art in volumetric image analysis. 3D CNNs are able to consider the 3-dimensional structure in a volume instead of operating slice by slice but require very large amounts of training data due to the large number of model parameters. Specifically, 3D CNNs have a substantially larger number of parameters compared to standard 2D CNNs. In addition, we also included a 2D CNN which used the same image tiling approach as SLIVER-net, which serves as a baseline model for assessing the effectiveness of transfer learning and slice pooling. The alternative deep learning models (see “Methods”) were trained to predict biomarkers associated with AMD using the same training data from the University of Miami and Case Western Reserve University (see “Methods” for more details about the train and test sets).

**Fig. 1:**
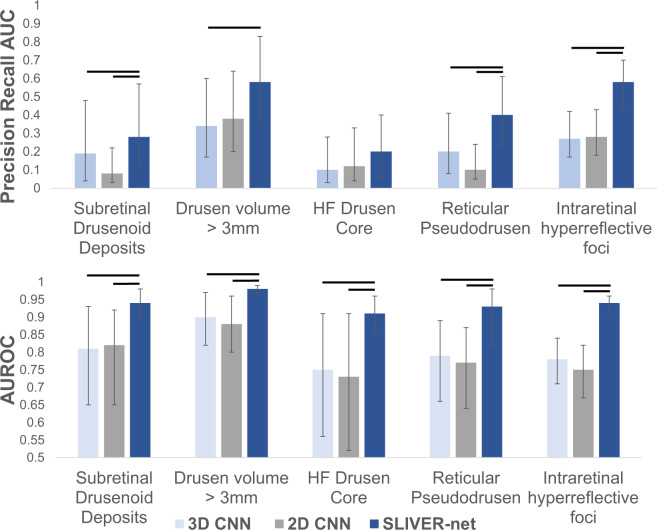
SLIVER-net performance. SLIVER-net (dark blue) was compared with a 3D-CNN backbone approach (light blue) and 2D CNN (gray). SLIVER-net significantly outperformed both the 3D CNN and 2D CNN in identifying each biomarker in terms of area under the ROC (AUROC) and area under the precision-recall curve (precision-recall AUC). Top: Precision-recall AUC for each biomarker. Bottom: ROC AUC for each biomarker. Horizontal bars indicate a significant difference in performance between the two models. Error bars represent 95% confidence interval (CI) calculated using a bootstrapping procedure.

Due to the strongly imbalanced nature of biomarker prevalence, the models were evaluated using area under the ROC curve (AUROC) and precision-recall curve (AUPRC) metrics. On the University of Pennsylvania test set (740 volumes), the 3D CNN predicted all biomarkers with a mean ROC area under the curve (AUC) of 0.81[95% confidence interval (CI): 0.75,0.86], and a mean precision-recall AUC of 0.22[CI: 0.17,0.33], and the 2D CNN performed with mean ROC AUC of 0.79[CI: 0.67,0.82] and a mean precision-recall AUC of 0.19[CI: 0.16,0.28]. SLIVER-net achieved a mean ROC AUC of 0.94[CI: 0.91,0.96], and a mean precision-recall AUC of 0.41[CI: 0.34,0.51] thus showing significant improvement over the alternative approaches in terms of ROC AUC (*p*-value < 0.001) and precision-recall AUC (*p*-value < 0.001). The performance of each individual biomarker is shown in Fig. 1.

### Comparison of SLIVER-net with specialist clinician assessments

In addition, we compared SLIVER-net’s predictions against expert retinal image graders (retina specialists who had been certified for OCT image grading by the Doheny Image Reading Center) with respect to the manually annotated biomarkers. Within the test set from University of Pennsylvania, 100 patients were randomly selected and their OCT volumes were read by an additional three retina specialists.

**Fig. 2:**
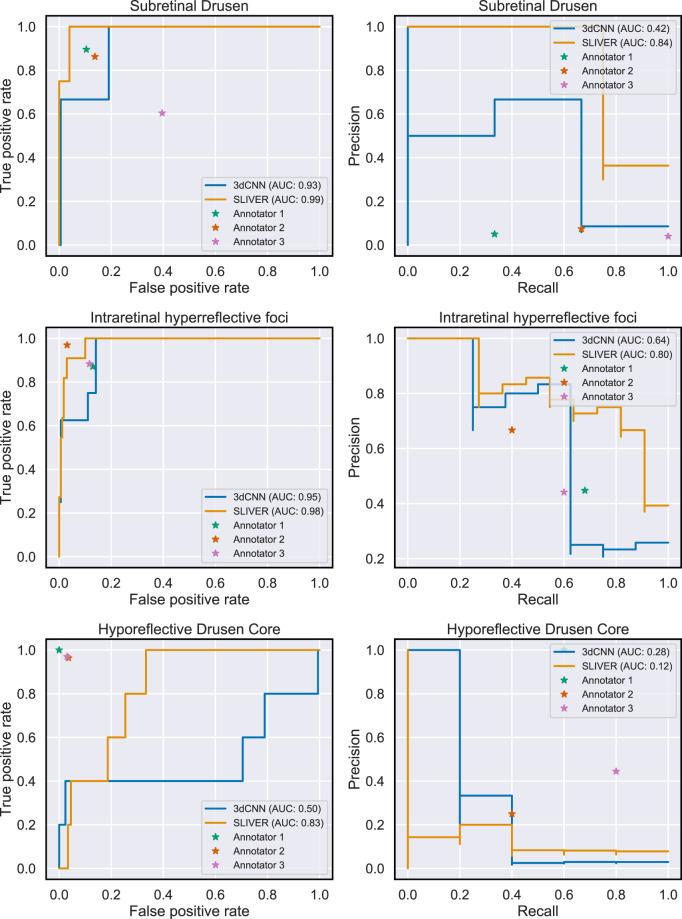
Comparison of model with clinicians. Our model identified three biomarkers that were annotated by clinicians. We present ROC (left column) and precision-recall (right column) curves for SLIVER-net and the baseline 3d CNN model along with individual annotator performance. For subretinal drusenoid deposits, SLIVER-net appears to outperform retina fellows in terms of both AUC and precision-recall, while the reverse is true for hyporeflective drusen cores.

**Fig. 3:**
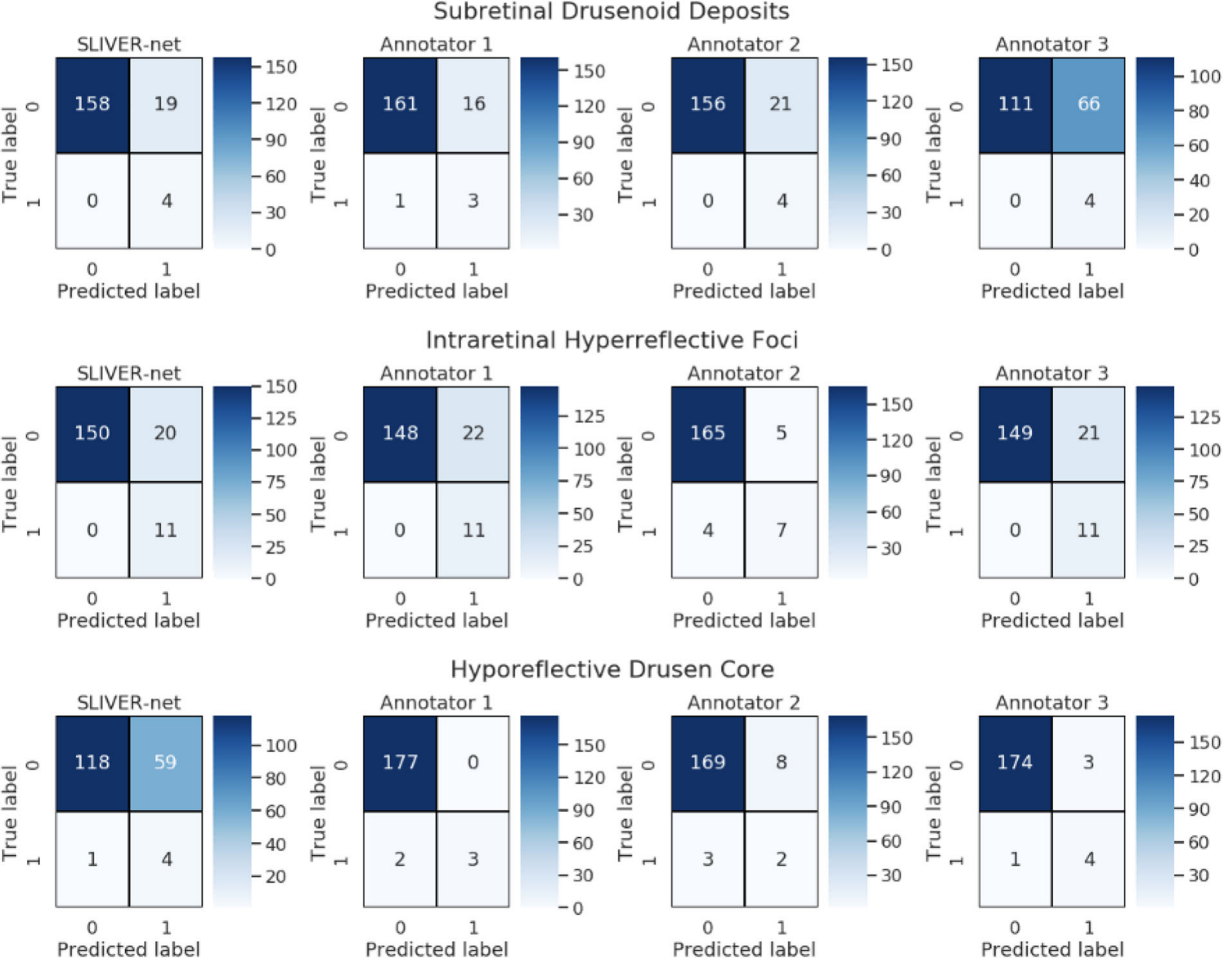
Confusion matrices for SLIVER-net and the three retinal specialist annotators. 100 of the 390 test set patients were selected for comparison with clinician performance. The remaining 290 patients were used to compute the SLIVER-net threshold, which was selected to match the mean sensitivity of the annotators. For Subretinal Drusenoid Deposits and Intraretinal HRF, SLIVER-net displays a similar sensitivity to clinicians while operating at fewer false positives.

We observed that SLIVER-net outperformed all clinician experts in identifying subretinal drusenoid deposits, two out of the three clinicians in identifying intraretinal hyperreflective foci in terms of both ROC metrics and precision-recall (Fig. 2), generally predicting fewer false positives while maintaining the same sensitivity (Fig. 3). However, SLIVER-net was inferior in identifying hyporeflective drusen cores. We also observed that SLIVER-net was successful at predicting high central drusen volume and reticular pseudodrusen which clinicians would not be able to assess without additional equipment.

**Fig. 4:**
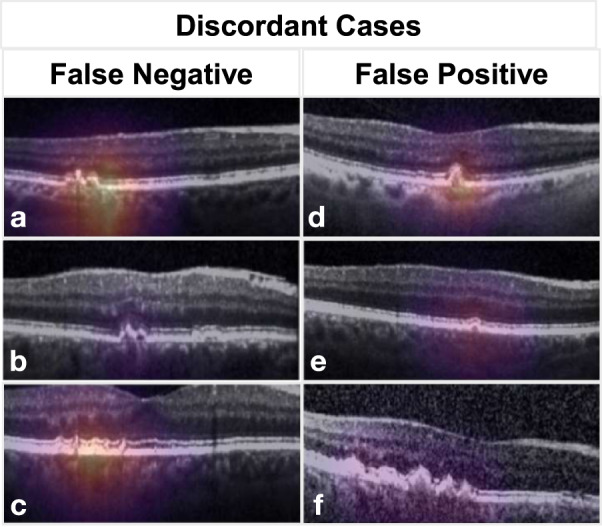
Examples of discordant cases. B-scans of example cases where SLIVER-net’s determination disagreed with the expert human graders, with heat map overlay highlighting the most informative regions of the image as determined by the algorithm. Panels **a**–**c** show examples of false-negative cases where the feature was detected by the grader on initial review, but not by the algorithm. In **a**, **b**, virtually no separation can be seen between retinal pigment epithelial (RPE) band and the drusen, which presumably made it difficult for the algorithm to determine that these were intraretinal hyperreflective foci (IHRF). In fact, on post hoc review, the senior retina specialist sided with the algorithm. In **c**, the heat map highlights the relevant features, but the algorithm failed to identify these tiny conical or spike-like elevations as subretinal drusenoid deposits (SDD). This was deemed to be a true false negative on post hoc review, yet it should be noted that no clear distinction in reflectivity is observed between the SDD and the underlying RPE. Panels **d**–**f** show examples of false positives where the algorithm detected a biomarker but the ground-truth human grader did not on initial review. In **d**, the heat map highlights a drusen but there are no apparent IHRF. However, there are occasional tiny bright dots in the Henle’s layer which are due to retinal capillaries but may have been confused as IHRF. This is a true false positive. In **e**, the algorithm detected a drusen with hyporeflective core (hDC), but the drusen was small and <40 µm in height. By definition, graders do not assess the internal reflectivity in lesions this small. The algorithm was able to make this assessment and the internal reflectivity is a bit reduced, but it is a true false positive as it does not match the grading convention. In **f**, the algorithm also determined hDC to be present, but the internal reflectivity of the drusen, while reduced, is not dark enough to be called hyporeflective. This is also a true false positive.

**Fig. 5:**
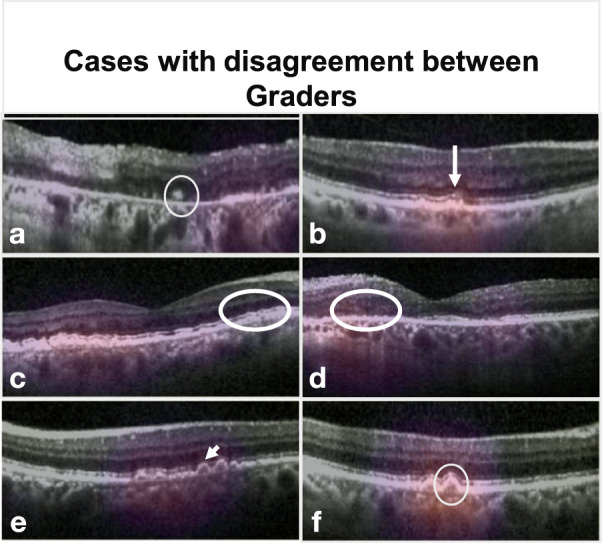
Composite of B-scan images of example cases with disagreement between multiple graders. Top row: IHRF, middle row: SDD, bottom row, hDC. In **a**, Aan IHRF is clearly visible (white circle) but is in a region of atrophy. Some graders excluded consideration of the feature as a result. This finding was correctly detected by the algorithm. **b** A tiny brighter dot (arrow) is observed in the ELM band. This was interpreted by some graders as a possible IHRF. However, the feature is too small and the reflectivity is not as bright as the RPE band. This finding was correctly excluded by the algorithm. **c**, **d** The EZ has a slightly “wavy” profile suggestive of possible underlying subretinal drusenoid deposits (within the white circles). In both these cases, the algorithm correctly identified the presence of these subtle SDD. **e** The drusen (white arrow) is relatively small and its height is borderline for being ≥40 µm, which is the minimum threshold set by the grading protocol in order to be able to assess internal reflectivity. Graders disagreed with regards to whether the lesion met the size criterion. **f** The internal reflectivity of the drusen is slightly reduced but is clearly brighter than the vitreous overlying the retina. The reflectivity is not sufficiently reduced to be confident that a hDC is present, and hence the disagreement between graders. IHRF intraretinal hyperreflective foci, ELM external limiting membrane, EZ ellipsoid zone, SDD subretinal drusenoid deposits, hDC hyporeflective drusen core.

Cases where SLIVER-net disagreed with specialist annotations were sent to the same clinician panel with an additional senior specialist for review (Table 1). The post hoc review revealed that most of SLIVER-net’s errors occurred during difficult reads, in which the biomarker was small, subtle, or located in close proximity to another structure which made it difficult to distinguish the feature from the background (e.g., a hyperreflective focus close to the RPE surface). In addition, there was disagreement among the clinician panel in many of the cases where SLIVER-net produced a false positive (Fig. 4). For subretinal drusenoid deposits, 16 of the 19 false positives (84.2%) did not have a consensus among annotators; for hyperreflective foci, 16 of the 20 false positives (80%) did not have a consensus; and for hyporeflective drusen core, 10 out of 59 false positives (16.9%) did not have a consensus from the annotators (examples are visualized in Fig. 5). After review, some of these false positives were deemed to be errors in the initial annotation, and in these cases SLIVER-net detected these biomarkers while the clinician panel did not (Fig. 4a, b). This further highlights the potential of SLIVER-net as an aid to clinicians in assessing for the presence of these biomarkers.

**Table 1:** Discordant cases were reviewed by a senior retina specialist grader (SS).

Post hoc analysis of discordant cases between algorithm and ground truth			
	Discordant after review	Concordant after review	Observations from post hoc review
IHRF FP (*N* = 5)	1	4	Small IHRFs could be observed but were close to the minimum threshold size to be included
IHRF FN (*N* = 4)	2	2	IHRF were in close proximity to the RPE band making separation from the band more difficult to discern
SDD FP (*N* = 10)	2	8	Poor quality of B-scan images makes it more difficult to separate the SDD from the outer retinal bands (EZ, RPE)
SDD FN (*N* = 7)	1	6	SDDs very small in size
hDC FP (*N* = 10)	5	5	Drusen of smaller size making assessment of internal reflectivity difficult. Level of hyporeflectivity was borderline
hDC FN (*N* = 1)	0	1	Feature missed by grader

Upon re-review the senior retina specialist disagreed with the original ground-truth grading in some cases, but in all discordant cases the findings were borderline. Observations with regards to the cause for difficulty in ground-truth assessment are provided.

*FP* false positive, *FN* false negative, *IHRF* intraretinal hyperreflective foci, *SDD* subretinal drusenoid deposits, *hDC* hyporeflective drusen core, *RPE* retinal pigment epithelium.

### Effect of sample size on the model performance

**Fig. 6:**
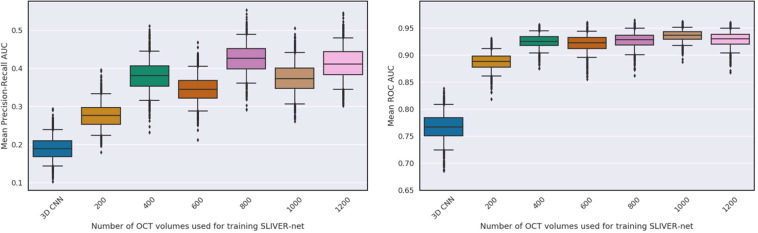
The effect of sample size on SLIVER-net’s performance. 3D CNN was trained on full data. Top: Mean precision-recall AUC across all biomarkers. Bottom: Mean ROC AUC across all biomarkers. Error bars represent 95% confidence interval (CI) calculated using a bootstrapping procedure.

We found that SLIVER-net outperforms a standard 3D CNN in the setting of a relatively small sample size. However, the necessary number of annotated samples required to achieve high performance is unclear. To address this question, we re-trained SLIVER-net with a reduced number of OCT volumes available and measured the performance on the test set (Fig. 6). We observed that a sample size of 200 OCT volumes was sufficient for SLIVER-net to achieve a mean ROC AUC of 0.89[CI: 0.86,0.92] and a mean precision-recall of 0.25[CI: 0.22,0.34], which is significantly better than the standard 3D CNN trained on the entire 1202 OCT volumes available in our training cohort (mean ROC AUC 0.81[CI: 0.75,0.86]). With a sample size of 400 volumes, SLIVER-net achieved a mean ROC AUC of 0.93[CI: 0.90,0.95] and a mean precision-recall of 0.36[CI: 0.30,0.46] which is not significantly different from its top performance, which, as previously shown, was at the level of expert retina graders. In this case, SLIVER-net was able to achieve the state-of-the-art and expert-level performance with a sample size three times smaller than the one collected for this work.

### Identifying traces of biomarkers outside of the macula

One advantage of deep learning is its ability to detect patterns without the usage of handcrafted features when given a sufficient amount of labeled data. In some cases, it is possible to annotate an object using one source, then train a model on a different one allowing the network to discover patterns unknown to researchers. This operation is useful when the information exists in the data but is unidentifiable by a human specialist.

In current practice, infrared reflectance (IR) imaging is commonly used to identify reticular pseudodrusen (RPD). RPD are now known to correspond to the subretinal drusenoid deposits which can be observed on OCT. Unlike IR images whose field of view is usually 30° or larger, OCT volumes obtained in clinical practice are commonly limited to a 6 × 6 mm (~20°) macular region centered on the fovea. RPD, however, are more frequently found in the more peripheral portions of the posterior pole outside of this 6 × 6 mm macular region. As a result, these lesions will not be identified on review of the OCT alone, thus potentially leading to an underestimation of the risk of progression to late AMD in these individuals. To determine if this limitation could be overcome, we took advantage of companion IR images available with the OCT volumes in the Amish dataset and labeled these IR images for the presence of RPD. SLIVER-net successfully predicted the presence of RPD with an ROC AUC of 0.93[CI: 0.82,0.98] and precision-recall AUC of 0.40[CI: 0.22,0.61], significantly better than chance, using the OCT scans limited to the macula. This suggests the existence of patterns available in OCT scans that are still unknown to human specialists.

### Transfer learning improves model performance

Our training data consisted of 1202 annotated 3-dimensional volume images for biomarker prediction. Among these volumes, the prevalence of biomarkers ranged between 2 and 8 percent (see Table 1: “Methods”), while deep learning models generally require many more. A key component of SLIVER-net was flattening the OCT volume into an image by stacking the different slices into one long image (see “Methods”). This allowed us to incorporate a large publicly available dataset^4^ using transfer learning, which is commonly used to address prediction problems when the amount of training data is small^17^. Under this paradigm, the model is “pre-trained” on a similar task, usually with a larger dataset. The model is then fine-tuned for the task at hand (see “Methods” for details).

SLIVER-net was pre-trained on the OCT dataset collected by Kermany et al.^38^. This data consisted of 84,495 2D horizontal OCT B-scan images (e.g., slices) passing through the fovea but were labeled with other ocular diseases (Choroidal neovascularization (CNV), diabetic macular edema (DME), and Drusen). The pre-trained network was then fine-tuned for the biomarker prediction task.

**Fig. 7:**
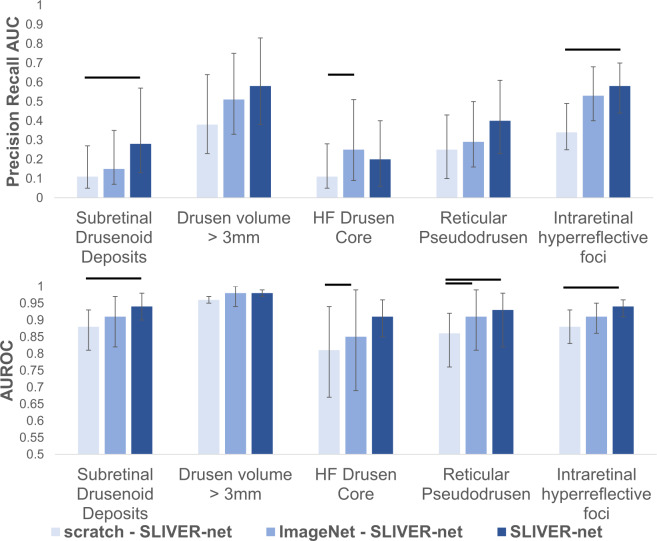
Transfer learning evaluation. Comparison of external datasets and their effect on performance. SLIVER-net trained from scratch (light blue), pre-trained using ImageNet (blue), and pre-trained using Kermany (dark blue). Top: Precision-recall AUC for each biomarker. Bottom: ROC AUC for each biomarker. Horizontal bars indicate statistically significant differences (*p* < 0.05). Error bars represent 95% confidence interval (CI) calculated using a bootstrapping procedure.

We evaluated the performance of SLIVER-net with and without pretraining. Pretraining the model with the Kermany data (reported above) resulted in significantly (*p* < 0.001) better performance when compared with training the model from scratch (mean ROC AUC 0.88[CI: 0.83,0.92]), mean precision-recall AUC 0.24[0.20,0.33], Fig. 7).

### The tradeoff between quantity and quality of external data

The effectiveness of the transfer learning procedure depends on the size of the external data, as well as its similarity to the target task. While the Kermany data above contained nearly 85,000 OCT scans, there are even larger but less related datasets. Natural images from the ImageNet^18^ dataset (over 1 million samples with 1000 classes) may provide a good foundation for the transfer learning approach based on the sheer volume of training data. We thus compared the performance of SLIVER-net pre-trained with data from Kermany et al. (Kermany-SLIVER) against the same model pre-trained with data from ImageNet (ImageNet-SLIVER). Kermany-SLIVER outperformed ImageNet-SLIVER with a mean ROC AUC of 0.94[CI: 0.91,0.96], and a mean precision-recall AUC of 0.41[CI: 0.34,0.51] compared with 0.92[CI: 0.87,0.95] (*p* < 0.01) and 0.35[CI: 0.30,0.45], respectively (see Fig. 7), despite the difference in the number of exemplars. This is in line with recent findings^39^ that while training set size is essential, it is beneficial in terms of performance to pre-train networks using related data.

### Robustness to the number of slices available in each volume

OCT acquisition parameters are not standardized in current ophthalmic practice. Notably, retina practitioners may determine the number of slices (B-scans) to acquire on a patient-by-patient basis, resulting in volumes with differing resolution and field of view. While the data acquired in this study were of the same resolution and field of view, we simulated scans of different field of view and resolution in order to assess SLIVER-net’s robustness to such changes.

**Fig. 8:**
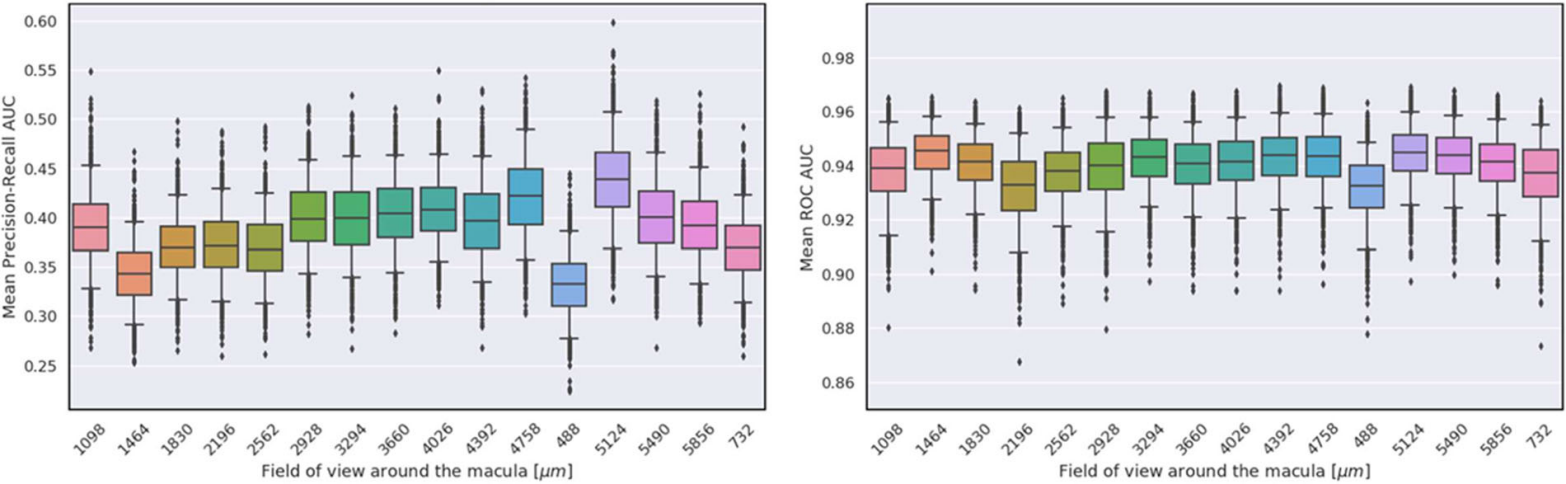
The effect of the field of view around the macula on SLIVER-net’s performance. Top: Mean precision-recall AUC across all biomarkers. Bottom: Mean ROC AUC across all biomarkers. Error bars represent 95% confidence interval (CI) calculated using a bootstrapping procedure.

**Fig. 9:**
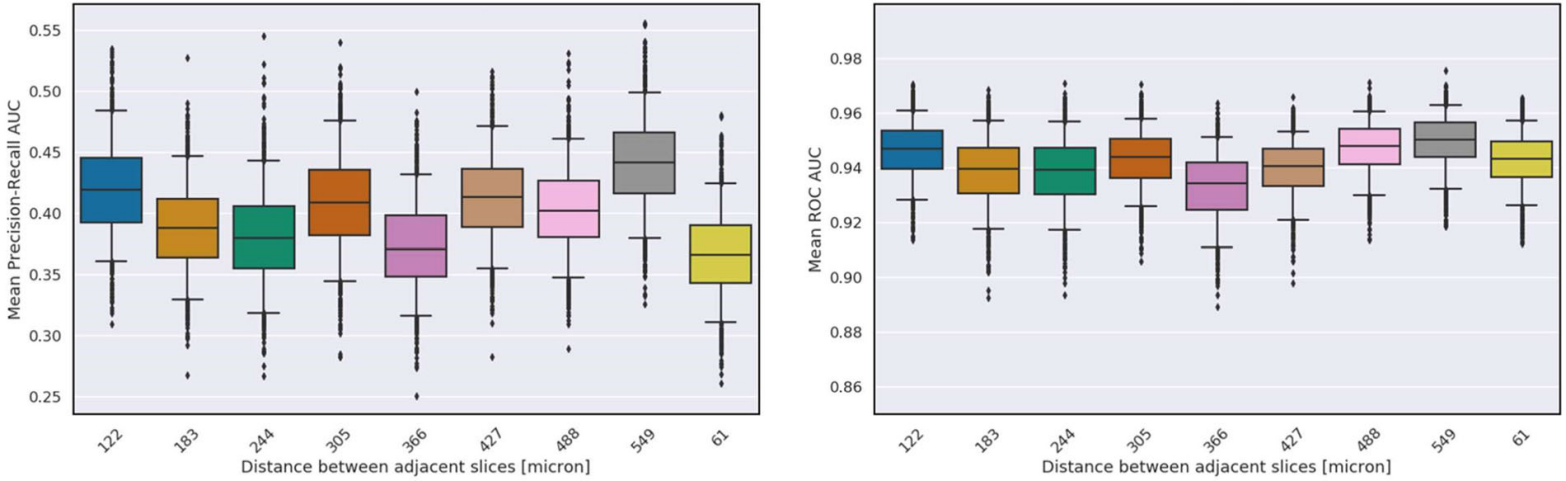
The effect of the resolution along the Z-axis on SLIVER-net’s performance. Top: Mean precision-recall AUC across all biomarkers. Bottom: Mean ROC AUC across all biomarkers. Error bars represent 95% confidence interval (CI) calculated using a bootstrapping procedure.

First, we artificially varied the field of view around the macula available in each volume (see “Methods”) and observed that varying the field of view did not significantly affect performance for biomarker prediction (Fig. 8). Then, we simulated different B-scan resolutions by down-sampling the number of slices in each volume (see “Methods”), again observing that varying volume resolution did not significantly affect the model’s performance for biomarker prediction (Fig. 9). In both scenarios, we have observed that SLIVER-net was robust to different sizes and resolutions of OCT scans, making it useful in various clinical scenarios and under different resource constraints.

## Discussion

The application of deep learning to new studies depends on the ability to train models with limited data. In this work, we developed a new deep learning technique, SLIVER-net, to predict clinical features from OCT volumes. Our approach provides these predictions using a relatively small number of annotated volumes (hundreds), and an even smaller number of positive training examples. SLIVER-net is based on two main ideas. First, we use transfer learning to borrow information about the structure and parameters of the network from publicly available large datasets. Unfortunately, there are no large datasets that include volumes, and we, therefore, use transfer learning using the 2D images. In order to account for this, our second idea is to model the volume as a 2-dimensional image by tiling the volume scans, and then adding to the neural networks additional layers that take into account the fact that two adjacent images in the tiled image are adjacent in the original 3D volume.

We demonstrate our approach using OCT volumes, which are widely used in current ophthalmic practice. Specifically, we used SLIVER-net to identify clinically useful OCT biomarkers which have been shown to predict the risk for progression to late AMD^34^. We found that for most features, SLIVER-net was able to identify these AMD-related biomarkers in agreement with senior expert clinician graders and was superior to junior graders. In many cases, as revealed by a post hoc review, SLIVER-net identified additional biomarkers that were missed by the initial annotation. SLIVER-net is considerably more powerful than standard deep learning techniques used for medical volumes such as 3D CNNs. Despite having very few annotated samples from our original dataset, SLIVER-net was able to outperform the current state-of-the-art methods. Particularly, our approach significantly improved the average AUC from 0.81 achieved by 3D CNNs to 0.94 achieved by SLIVER-net. The models were compared using an external test set acquired at a separate institution, which, in contrast with single-site and single-dataset studies, provides support that SLIVER-net can be portable across institutions.

At a practical level, SLIVER-net provides a general framework for addressing prediction problems with a limited sample size of labeled data. Its success was primarily driven by transfer learning and slice integration, both of which are not limited to biomarker prediction nor OCT classification. Thus SLIVER-net presents a feasible approach to the application of deep learning to new problems involving 3-dimensional imaging modalities. While typical machine learning solutions cite requirements in the tens of thousands in terms of training samples, our investigations showed that SLIVER-net approached maximum performance with only 400 training samples (Fig. 8), which more closely matches sample sizes required for clinical validation. Using the transfer learning framework, predictive and data-driven applications can potentially be pursued concurrent to clinical validation without devoting additional resources to annotation.

The early application of deep learning and automated image analysis to relatively new imaging modalities such as OCT can also provide a synergistic development at technical and clinical levels. We included reticular pseudodrusen (RPD) as a biomarker of interest because it is a lesion which may in some cases be present only beyond the typical macular OCT scanning field commonly used in clinical practice, and is thus instead detected using larger field of view infrared reflectance imaging. Interestingly, SLIVER-net was able to successfully detect the presence of RPD using the smaller field macular OCT information alone, which suggests that lesions which fall outside the macula may be associated with subtle alterations in the macula which remain to be understood. Future work utilizing multiple imaging modalities that are available for use in clinical practice, may reveal other novel findings which may be encoded in the OCT data.

The ability to automatically identify these high-risk biomarkers for AMD progression has important clinical implications. Lei et al.^12^ have already shown that the presence of these biomarkers can be translated to a simple score that can risk stratify patients presenting to the clinic. Automated biomarker detection could lead to a more precise quantification of not only the presence but the extent or severity of the biomarker or feature of interest, which could further improve the predictive accuracy of the biomarker^40^. Such a risk score could be used to prognosticate disease and to define appropriate intervals for follow-up and monitoring. This is particularly relevant as home OCT devices are now becoming available for telescreening. In addition, such a scoring system could also be used to identify high-risk patients for enrollment in clinical trials for early intervention therapies. Automated biomarker detection could also prove to be invaluable in a number of research applications such as the study of the appearance and evolution/progression of these biomarkers in large AMD datasets. Investigations such as this may provide new insights into the pathogenesis of AMD.

## Methods

### Data

#### Biomarker prediction data

OCT scans were acquired from 1007 patients as part of a longitudinal study on AMD progression in an elderly Amish population. These scans were acquired from three different sites: University of Pennsylvania (390 patients), Case Western Reserve University (248 patients), and University of Miami (369 patients) using the Spectralis system (Heidelberg Engineering). The research was approved by the institutional review boards (IRBs) of the respective institutions and all subjects signed written informed consent. All research was conducted in accordance with the tenets set forth in the declaration of Helsinki. All imaging data were transferred to the Doheny Image Reading Center (DIRC) in a de-identified fashion. The image analysis research was approved by the UCLA IRB. Two volumes (97 B-scans, with an in-plane resolution of 496 × 512 and dimension of 6 × 6 mm on the retina—roughly a 20-degree field of view) were acquired from each patient. Only scans that were determined to be good quality, as assessed by a senior retina image grader at the Doheny Image Reading Center, were used for model development and validation. Under this criterion, we excluded 72 volumes, resulting in 1942 OCT volumes in total. Data from the University of Miami and Case Western Reserve University (1202 volumes) were used for model training, and data from the University of Pennsylvania (740 volumes) were withheld for testing.

Four biomarkers (hyperreflective foci, hyporeflective cores within drusen, subretinal drusenoid deposits, and high central drusen volume), and reticular pseudodrusen as identified using IR imaging, were selected for this study. A single retina specialist reviewed each Spectralis OCT volume, manually recording the presence of hyperreflective foci, hyporeflective drusen cores, and subretinal drusenoid deposits. The remaining two biomarkers were identified using different devices. The Cirrus OCT system (Zeiss) was used to quantify central drusen volume, and reticular pseudodrusen were identified using an infrared reflectance image. In accordance with previous publications^12,34^, a high central drusen volume was determined to be a value of ≥0.03 mm^3^ within the central 3 mm zone centered on the fovea. It is important to emphasize that the Spectralis system cannot produce a drusen volume measurement, though the drusen are visible on the OCT. In addition, while subretinal drusenoid deposits (SDD) evident on OCT appear to correspond to reticular pseudodrusen (RPD), RPD are commonly present only outside the macula, and thus RPD may be present on an IR image (which covers a 30-degree field of view) without evidence of visible SDD on the OCT. Table 2 summarizes the prevalence of these biomarkers within the dataset.

**Table 2:** The biomarkers used for this study and their prevalence throughout the three datasets.

	Training set	Testing set	Total
Number of patients	617	390	1007
Number of OCT volumes	1202	740	1942
Hyperreflective foci (IHRF)	89 (7.4%)	49 (6.6%)	138 (7.1%)
Hyporeflective drusen core (hDC)	33 (2.7%)	13 (1.8%)	46 (2.4%)
Subretinal drusenoid deposits (SDD)	23 (1.9%)	13 (1.8%)	36 (1.9%)
High central drusen volume	40 (3.3%)	19 (2.6%)	59 (3.0%)
Reticular pseudodrusen (RPD)	41 (3.4%)	20 (2.7%)	61 (3.1%)

The OCT volumes of 91 patients randomly selected from University of Pennsylvania were annotated by an additional three junior reading center clinician graders. These labels were used to assess inter-rater reliability as well as model comparison.

#### Transfer learning data

We compiled two external datasets to pre-train our models. One dataset was ImageNet^18^, which consists of millions of training images comprised of a thousand object categories. ImageNet has been commonly used in transfer learning applications for natural images, and it has been shown that models pre-trained on ImageNet perform well on other domains^41,42^.

We also acquired a large collection of publicly available OCT images collected by Kermany et al., which we simply refer to as “Kermany”. In this dataset, 84,495 horizontal B-scans passing through the foveal center (i.e., typically the middle slice of an OCT volume) were annotated for one of four conditions: normal, choroidal neovascularization (CNV), diabetic macular edema (DME), and drusen. While there were less than 100,000 samples in this dataset, they were more similar to our biomarker prediction data.

#### Data preprocessing

Each slice of the volume was resampled from 496 × 512 pixels to 224 × 224 pixels^43^. Then, image contrast was enhanced by clipping pixel intensities to the 2nd and 98th percentile, and resulting values were rescaled between 0 and 255.

### 3D CNNs

Convolutional neural networks (CNNs) comprise many kernels that receive an image as input and produce a representation that is most meaningful for a given task using an operation called convolution (see Supplementary Information for details). 3D CNNs extend this approach to three-dimensional objects and are commonly applied to volume analysis. They have gained popularity in biomedical imaging (e.g., CT^36,44^, MRI^37,45–47^) due to increasingly capable hardware. We used a 3D version of Resnet18^43^ (see Supplementary note for details) to compare against the 2D approach. The input to the network was a 3D volume of size 224 × 224 × 97 and the output was a prediction score range 0–1 for each biomarker representing the probability the respective biomarker is present. Detailed model parameters can be found in Supplementary Table 1.

### SLIVER-net architecture

Our proposed approach, termed SLIVER-net, was comprised of three steps. First, the preprocessed OCT volume was passed through a “backbone” convolutional neural network (CNN), which represented the scan in an abstract feature space. Then, a slice aggregation operation was applied in order to compress this representation and capture information that is shared across adjacent slices. Finally, a decision module operated on this compressed representation to determine the presence or absence of biomarkers.

#### Step 1: Backbone networks

CNN models contain several convolutional layers stacked together (i.e., each layer’s output serves as the next layer’s input) to extract a feature map from a single image. Previous work^48,49^ has shown that the first CNN layers (lower layers) of a deep learning model generally identify abstract features (lines, edges, corners) and the upper layers identify features that are more task-specific. In our experiments, all tested models were based on the same CNN architecture, Resnet18^43^ (see Supplementary Information for further details). 2D backbones (SLIVER-net) used 2D kernels (size 3 × 3, 7 × 7) while the 3D-CNN backbone used 3D kernels (size 3 × 3 × 3, 7 × 7 × 7). Resnet18 was chosen since it has shown to perform well in the natural image setting^43^. This model represents each 224 × 224 OCT slice as an 8 × 8 image.

**Fig. 10:**
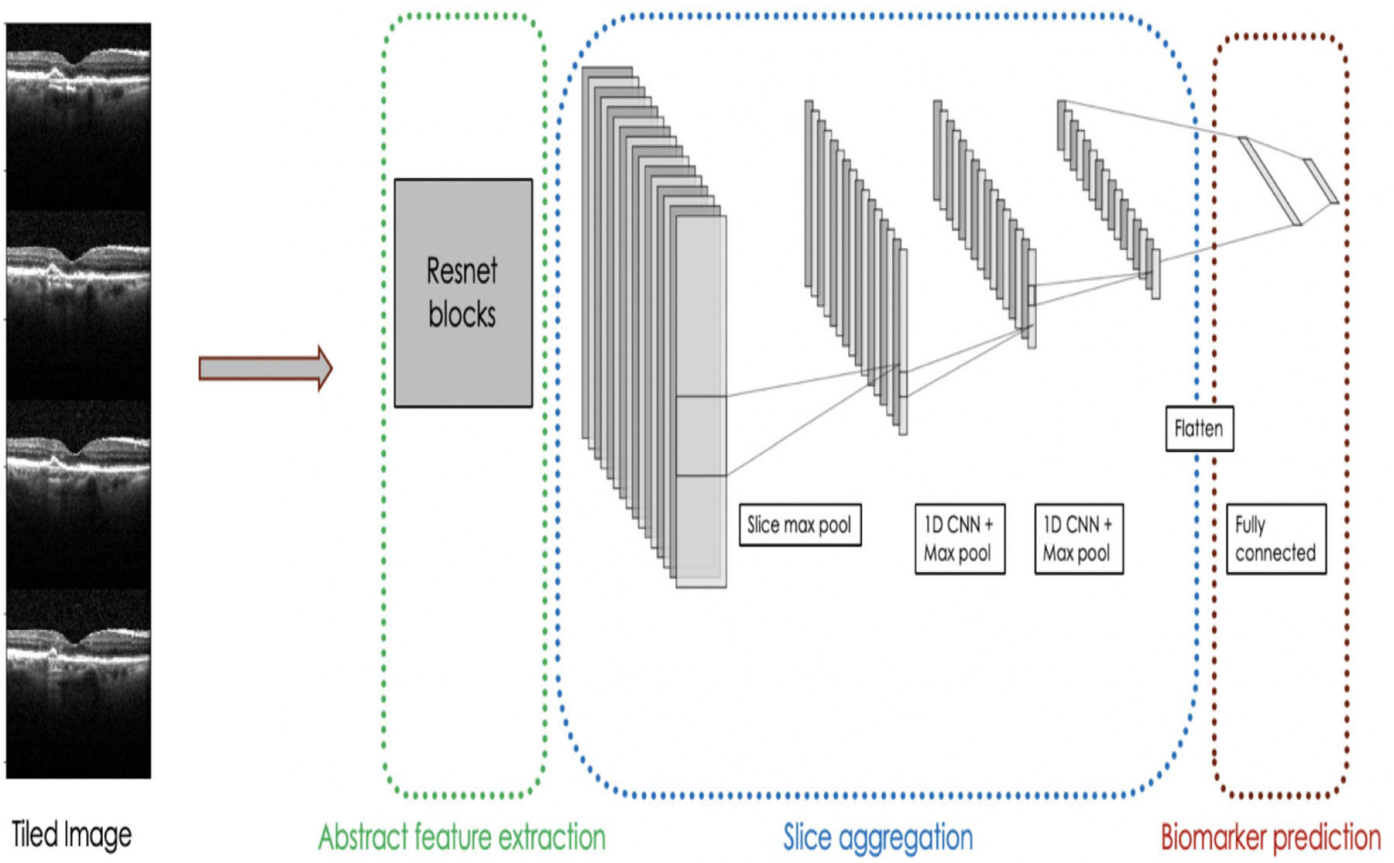
SLIVER-net. Our model operated on a 2d tiling of the OCT volume. Resnet18 served as the abstract feature extractor, and the representations for each slice were aggregated using slice integration and a 1D CNN. Finally, biomarkers were predicted using fully connected layers.

Feature extraction on all 2D slices was computed in one forward pass. To do this, each of the 97 slices was concatenated vertically, forming a “tiled” image of (97 × 224) × 224 (see Fig. 10) that was passed to the model. The output of the backbone model was a (97 × 8) × 8 image with 1024 features for each of the 97 slices.

#### Step 2: Slice integration

In a deep learning model, the final feature map produced by the CNN layers is collapsed into a feature vector, usually by taking the average across all spatial dimensions in an operation referred to as global average pooling^50^. This “flattens” the feature map such that it can be passed to a decision module. We extended this operation by taking both the maximum (“max pooling”) and average (“average pooling”).

However, we observed that applying this operation globally would remove the model’s access to the local 3D structure of the OCT volume. In order to preserve correspondence among neighboring slices, we performed average and max pooling within each of the 8 × 8 backbone outputs, producing a 97 × 1024 representation of the volume. Then, a small 1D CNN was added to aggregate these slices before they were passed to the decision layer. This slice integration procedure was a primary driver of the success of SLIVER-net.

#### Step 3: Decision module

Biomarkers were predicted in a multi-task approach, in which the single network simultaneously predicted the presence of all targets. Our prediction “head” consisted of only one hidden layer with 1024 hidden units, feeding to an output layer of 5 units with a sigmoid activation function, corresponding to the biomarkers. By simultaneously optimizing for separate tasks, the multi-task paradigm provides an implicit regularization, improving generalizability^51,52^.

### Training

Data acquired from the University of Miami and Case Western Reserve University were used to develop the models. These data were randomly split into training (80%) and validation (20%) sets. Models were implemented using PyTorch^53^ and optimized using the Adam optimizer with default parameters^54^ and a weight decay of 0.01. For each model, the learning rate was chosen from values between 1.0 and 10e−7 using the learning rate finder implemented in the Fastai library^55^. Models were trained with a batch size of 32, and training continued until validation loss stopped decreasing for 20 consecutive epochs (i.e., passes through the training dataset). The model weights that achieved the lowest loss on the validation set during training were chosen for evaluation on the test set.

### Transfer learning

One limitation of the Resnet and other CNN feature extractors is that they require a large amount of data to train. A typical solution to this is to apply transfer learning^16,17^, in which the network is first trained on an existing but similar dataset, and then “fine-tuned” on the dataset of study.

#### Model pretraining

We evaluated the ImageNet and Kermany datasets for their suitability for transfer learning. While ImageNet is a much larger dataset, the Kermany set, consisted of OCT images similar to our data.

The original labels for the candidate datasets (image classification for ImageNet, and disease diagnosis for Kermany), were not aligned with our biomarker prediction task. However, it has been observed^48^ that some convolutional neural networks extract general features applicable to most visual tasks. We used the following approach to apply transfer learning to biomarker prediction: (1) We trained a network for the original task of the auxiliary dataset. For both datasets, a Resnet18 feature extractor was trained for its respective task (object classification or disease classification) for up to 50 epochs (with early stopping) and a learning rate of 1e−3. (2) We discarded the decision layers, which were specialized for the auxiliary task, and (3) replaced the decision layer with a randomly initialized one appropriate for the target task. (4) Only the new decision layer for biomarker prediction was then trained with our training set without updating any of the parameters in the feature extractor. (5) Finally, the whole network was updated using a reduced learning rate of 1e−5.

### Model evaluation

Model performance on the test set was quantified in terms of the area under the receiver operating characteristic (ROC) curve, as well as the precision-recall (PR) curve. A 95% confidence interval was estimated for model performance using a bootstrapping procedure. For each bootstrap iteration, we randomly resampled from the test set with replacement and calculated performance metrics. We repeated this 5000 times and selected the 125th and 4875th values of the sorted list to define the 95% confidence interval. Performance metrics were compared using a Wilcoxon signed-rank test^56^ (i.e., nonparametric *t*-test).

### Model explainability

In clinical settings, it is of high importance for statistical models to communicate some rationale behind decision making in order to build trust between the machine learning algorithm and the clinical user. To address this issue, we provide explainability maps along predictions to show important regions as inferred by the algorithm (Supplementary Fig. 1). The explainability maps are produced by visualizing the backbone representation of each OCT volume. The representation of each slice (an 8 × 8 feature map with 512 channels) is averaged across channels to create an 8 × 8 feature image for each scan (97 total scans in each volume), which shows the average local importance across all channels. Then, the feature image is interpolated to match the sizes of the original input. The featured image and the original input are shown together to produce the explainability map.

### Simulating model performance with different acquisition parameters

We assessed the robustness of SLIVER-net to different acquisition parameters by artificially varying the OCT volumes. In each case, we trained SLIVER-net on the transformed data and observed performance on the test set with the same transformation.

To manipulate field of view, we used various numbers of slices taken around the macula. We evaluated performance when 9 central slices (488 microns) were available up to 97 slices (5856 microns). Then, to evaluate the SLIVER-net’s performance on the resolution of each volume along the Z-axis, i.e., the distance between two nearby slices, we used different sampling rates to down-sample the number of slices in each volume, thus simulating lower-resolution OCT volumes. We varied the distances between two nearby slices from 61 microns (97 slices total, the standard resolution of this study) up to a range of 549 microns between each (11 slices per volume).

### Reporting summary

Further information on research design is available in the Nature Research Reporting Summary linked to this article.

## Supplementary information

Supplemental Information

Reporting Summary

## Data Availability

The data are not publicly available due to institutional data use policy and concerns about patient privacy. However, the data that support the findings of this study are available on reasonable request from the corresponding author.

## Code availability

The analysis was conducted in Python, making extensive use of the Pytorch and Fastai packages. The analysis code is available at https://github.com/rakoczUCLA/sliver_net.
