# Supplementary material for: Automated identification of clinical features from sparsely annotated 3-dimensional medical imaging
Source: NPJ Digit Med. 2021 Mar 8;4:44. doi: 10.1038/s41746-021-00411-w (PMC7940637; doi:10.1038/s41746-021-00411-w)
Supplement: Supplementary file 1 — Supplemental Information [file 41746_2021_411_MOESM1_ESM.pdf]

Supplementary information for Automated identification of clinical features from sparsely annotated 3-dimensional medical imaging

Supplementary Analyses

Slice integration preserves 3D structure

To enable transfer learning, we first represent volumes as 2-dimensional tiled (e.g., “stacked”, see figure 1) images. We applied a standard deep neural network (Resnet18) to our proposed representation which we term Tile-RESNET, which was composed of the tiling approach outlined above combined with average and max pooling. By default, the convolution layers of the Resnet 18 produces a feature representation in the form of 512 "images," with the shape of 97x8 (an 8x8 image for each slice stacked on top of each other). Each of those images corresponds to an abstract feature learned by the network. The average and maximum were taken across the entire (97 x 8) x 8 backbone output, resulting in a vector of 1 x 1024 which was passed to the decision layer. However, in converting the 3D representation to 2D, a standard CNN (a RESNET18 for example) would lose the ability to capture 3-dimensional patterns.

In order to address this matter, we extended upon Tile-RESNET, by adding a Slice Integration module, consisting of a 1D CNN that encouraged the model to respect the local spatial structure and utilize the original volumetric shape of our generated 2D tile. SLIVER-net is thus the combination of Tile-RESNET with Slice Integration. We compared SLIVER-net to Tile-RESNET where both models were pre-trained using transfer learning to evaluate Slice Integration.

Tile-RESNET predicted AMD-related biomarkers with a mean ROC AUC of 0.89[CI: 0.84,0.93] and a mean precision-recall AUC of 0.38[CI: 0.32,0.48]. Outperformed by SLIVER-net ($p < 0.001$) in terms of the mean ROC AUC, suggesting that the addition of Slice Integration improved the overall performance due to SLIVER-net’s ability to identify 3-dimensional patterns (Figure S2).

Supplementary methods

Deep convolutional neural networks

Deep convolutional neural networks comprise out of many kernels that extract from every image the features that are most meaningful for a given task using a process called convolution. A convolution between an image and a kernel is very similar to correlation in that the image is scanned for patches (usually 3 x 3 pixels) that resemble the kernel. The output of a convolution layer is an image where the value of each pixel is the inner product between the corresponding patch in the input image and the kernel where patches that resemble the kernel produce high values and ones that do not produce values close to zero. In a deep network, the output Image of each convolutional layer is the input to the next one where at the end of the deep network the

final output is an abstract representation of the image. The idea behind deep convolutional neural networks is to make the network learn the different kernels that will extract meaningful shapes and remove ones that are destructive in order to learn a representation that is optimal for a given task.

Decision module

A decision module receives a feature vector as input and produces an output score in the range of 0 to 1 for classification or real value for regression problems. Our Decision module is a multilayer perceptron (MLP)[29–31]consisting of three fully connected layers with a rectified linear unit (ReLU)[32]between each layer and a sigmoid activation function as output. The fully connected layers which are linear operations combined with the nonlinearity of the ReLU functions enable the MLP to perform as a universal approximator[31]being able to approximate any function given a sufficient amount of data.

Demographic Covariates.

SLIVER-net is purely an image-based model, and does not consider other factors such as sex and age. We found that adding sex and age information to the model did not significantly improve performance, although age did appear to be predictive of some biomarkers. This suggests that SLIVER-net captures age information to some degree, despite not being explicitly trained to do so.

Supplementary Tables and Figures

Supplementary Table 1. Summary of candidate models for biomarker prediction. The input to each model was the same set of OCT images. However, they were represented differently (3D vs 2D) and passed through different subsequent layers with implications for spatial representation. Rows, Columns, and Channels represent the dimension of the abstract feature extraction of each volume at the output of the CNN backbone.

	3D CNN	SLIVER-net
Preprocessing	Slicing and contrast enhancement	
	97 slices of 224 x 224 pixels	
Abstract feature extraction	3D Resnet18	2D Resnet18
	8 x 8 x 4 x 1024	(97 x 8) x 8 x 512
	Rows = 8	Rows = 97 x 8
	Columns = 8	Columns = 8
	Channels = 8 x 1024	Channels = 512
Slice aggregation	3D Global Adaptive average pooling	Slice-Integration (local pooling + 1D CNN)
	1 x 1024	12 x 32
Biomarker prediction	Flattening + Fully connected	
Number of parameters	15,012,806	11,245,086

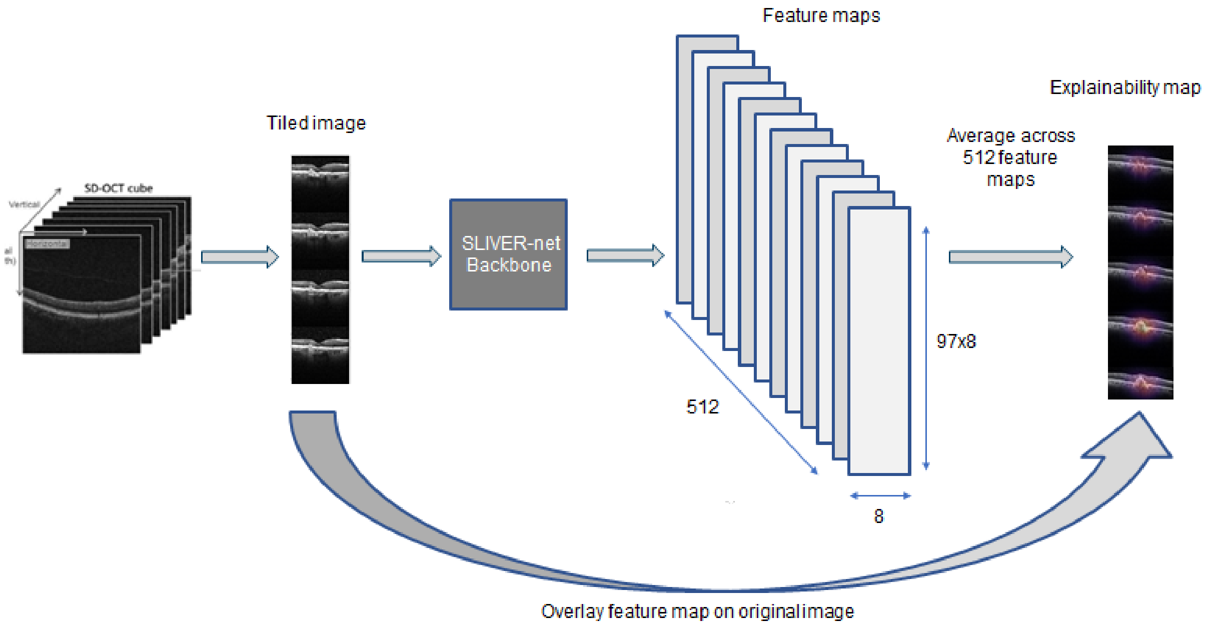

Supplementary Figure 1. Generating explainability maps from an OCT volume. Feature maps are generated by the Resnet blocks which form SLIVER-net's backbone. These feature maps are averaged across channels and resampled to the original size to visualize the image importance.

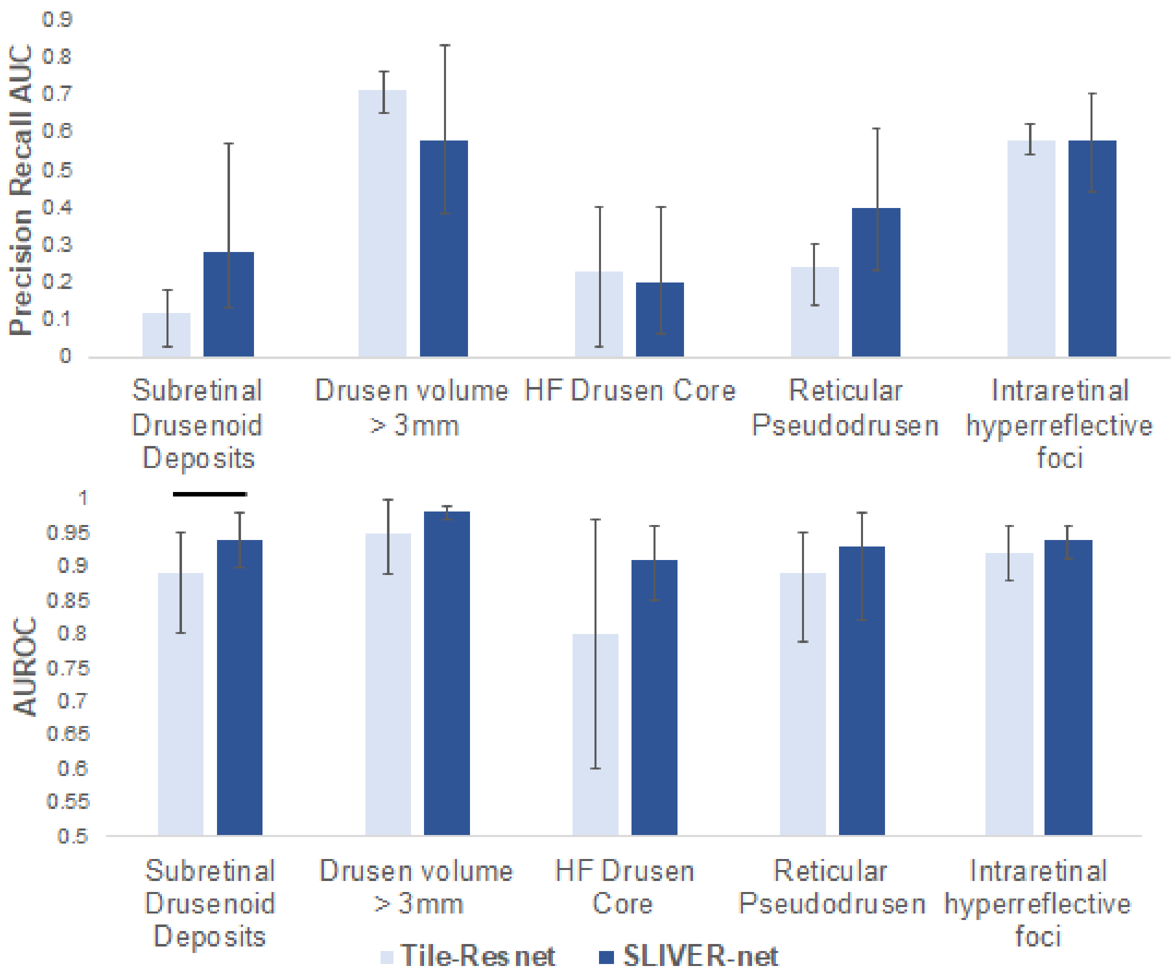

Supplementary Figure 2. Evaluation of the Slice-Integration operation. Biomarker prediction without (light blue) and with (dark blue) the Slice-Integration operation. Top. Precision-Recall AUC for each biomarker. Bottom. ROC AUC for each biomarker. Horizontal bars indicate a significant difference in performance between the two models. Error bars represent 95% confidence interval (CI) calculated using a bootstrapping procedure.
